# Flavopiridol Pharmacogenetics: Clinical and Functional Evidence for the Role of SLCO1B1/OATP1B1 in Flavopiridol Disposition

**DOI:** 10.1371/journal.pone.0013792

**Published:** 2010-11-01

**Authors:** Wenjun Ni, Jia Ji, Zunyan Dai, Audrey Papp, Amy J. Johnson, Sunjoo Ahn, Katherine L. Farley, Thomas S. Lin, James T. Dalton, Xiaobai Li, David Jarjoura, John C. Byrd, Wolfgang Sadee, Michael R. Grever, Mitch A. Phelps

**Affiliations:** 1 Division of Pharmaceutics, College of Pharmacy, The Ohio State University, Columbus, Ohio, United States of America; 2 Comprehensive Cancer Center, The Ohio State University, Columbus, Ohio, United States of America; 3 Department of Pharmacology, College of Medicine, The Ohio State University, Columbus, Ohio, United States of America; 4 Division of Hematology and Oncology, The Ohio State University, Columbus, Ohio, United States of America; 5 Division of Biostatistics, College of Public Health, The Ohio State University, Columbus, Ohio, United States of America; Dr. Margarete Fischer-Bosch Institute of Clinical Pharmacology, Germany

## Abstract

**Background:**

Flavopiridol is a cyclin-dependent kinase inhibitor in phase II clinical development for treatment of various forms of cancer. When administered with a pharmacokinetically (PK)-directed dosing schedule, flavopiridol exhibited striking activity in patients with refractory chronic lymphocytic leukemia. This study aimed to evaluate pharmacogenetic factors associated with inter-individual variability in pharmacokinetics and outcomes associated with flavopiridol therapy.

**Methodology/Principal Findings:**

Thirty-five patients who received single-agent flavopiridol via the PK-directed schedule were genotyped for 189 polymorphisms in genes encoding 56 drug metabolizing enzymes and transporters. Genotypes were evaluated in univariate and multivariate analyses as covariates in a population PK model. Transport of flavopiridol and its glucuronide metabolite was evaluated in uptake assays in HEK-293 and MDCK-II cells transiently transfected with SLCO1B1. Polymorphisms in *ABCC2*, *ABCG2*, *UGT1A1*, *UGT1A9*, *and SLCO1B1* were found to significantly correlate with flavopiridol PK in univariate analysis. Transport assay results indicated both flavopiridol and flavopiridol-glucuronide are substrates of the SLCO1B1/OATP1B1 transporter. Covariates incorporated into the final population PK model included bilirubin, SLCO1B1 rs11045819 and ABCC2 rs8187710. Associations were also observed between genotype and response. To validate these findings, a second set of data with 51 patients was evaluated, and overall trends for associations between PK and PGx were found to be consistent.

**Conclusions/Significance:**

Polymorphisms in transport genes were found to be associated with flavopiridol disposition and outcomes. Observed clinical associations with *SLCO1B1* were functionally validated indicating for the first time its relevance as a transporter of flavopiridol and its glucuronide metabolite. A second 51-patient dataset indicated similar trends between genotype in the *SLCO1B1* and other candidate genes, thus providing support for these findings. Further study in larger patient populations will be necessary to fully characterize and validate the clinical impact of polymorphisms in *SLCO1B1* and other transporter and metabolizing enzyme genes on outcomes from flavopiridol therapy.

## Introduction

Flavopiridol (Alvocidib, NSC 649890), is a serine/threonine kinase inhibitor that broadly targets cyclin-dependent kinases (CDKs), including the cyclin 9/cyclin T complex (pTEF-b), preventing activation of RNA polymerase II [1]–[2]. Flavopiridol initiates cell cycle arrest [3],[4] and p53-independent apoptosis [5]–[6] through down-regulation of Mcl-1 and X-linked inactivator of apoptosis (XIAP) [7], [8] [9]. These preclinical characteristics provided the rationale for clinical investigation of flavopiridol in chronic lymphocytic leukemia (CLL), as advanced CLL is commonly associated with elevated Mcl-1 and dysfunctional p53, rendering standard treatments such as alkylating agents, fludarabine and rituximab ineffective [10].

Single agent flavopiridol administered with 72-, 24- and 1-hour infusion schedules produced limited activity in hematologic and solid tumor diseases [11], [12], [13], [14]. Phase I and II studies using flavopiridol in combination with other agents utilizing the various schedules obtained mixed results, although partial and complete responses in these trials indicated potential synergy of flavopiridol with chemotherapy [15]. We previously reported overall response rates of 40–50% in patients with refractory CLL when flavopiridol was administered as a single agent using a pharmacokinetically (PK)-directed schedule [16], [17]. A phase II registration trial is underway for unmet need in refractory CLL patients using this PK-directed schedule.

The activity of the PK-directed schedule in CLL, compared to that of the previously evaluated schedules, clearly indicted the importance of flavopiridol PK for clinical activity, and associations were in fact observed between PK and clinical outcomes, including response, cytokine release syndrome (CRS) and tumor lysis syndrome (TLS) [17]. However, a substantial amount of variability in PK, as well as in response and toxicity, was unexplained by demographic, patient and disease characteristics. We therefore sought to determine the role of pharmacogenetic factors in flavopiridol PK and treatment outcomes within this patient population.

Flavopiridol elimination occurs via excretion and metabolism and is known through *in vitro* studies to be influenced by the multi-drug resistance protein-2 (MRP2, ABCC2) [18], [19] and the breast cancer resistance protein (BCRP, ABCG2) [20], [21], [22], [23], [24], which contribute to biliary excretion of both parent drug and glucuronide metabolites. Glucuronide conjugation to the 5- and 7-hydroxy positions of flavopiridol by uridine diphosphate glucuronosyltransferase isoforms 1A1 and 1A9 (UGT1A1 and UGT1A9, respectively) accounts for the majority of metabolic transformation of flavopiridol [25], [26]. Polymorphic diversity in these and other genes may influence flavopiridol disposition, activity and toxicity in a manner similar to irinotecan disposition [27], [28]. Limited polymorphism effects on flavopiridol interactions have been reported, including a lack of observed effects on clinical PK [29] and *in vitro* substrate specificity [30]. Although polymorphisms were not directly evaluated by Innocenti and colleagues, their clinical report suggested flavopiridol∶metabolite ratio as a possible predictor of diarrhea with flavopiridol treatment and provided a rationale for evaluation of a genetic link with UGT isoforms [31].

In this report, we present pharmacogenetic (PGx) data for drug metabolizing enzymes and transporters (DMET) in a subset of 35 patients treated in a phase I study of a PK-derived 4.5-hour dosing schedule of single-agent flavopiridol in relapsed CLL. These data comprise a focused analysis of candidate genes known through *in vitro* studies to interact with flavopiridol, as well as a broader exploratory evaluation of additional DMET genes. The results indicate a novel link between flavopiridol PK and SLCO1B1, as well as functional evidence for OATP1B1 (the organic anion transporting polypeptide 1B1 protein product of SLCO1B1) transport of flavopiridol and its glucuronide metabolite. Preliminary analysis of these associations in a second dataset comprising 51 patients provided additional support for the validity of associations between PK, transporter and UGT1A1 genes and their potential clinical relevance. Importantly, pharmacogenetic factors explain a significant portion of inter-patient variability and improves the accuracy of a developing population PK model for this agent.

## Methods

### Patients

#### Ethics Statement

Samples were obtained from patients who provided informed written consent and were enrolled on clinical protocol NCI-5746 (NCT00058240, ClinicalTrials.gov). The sample collection and analyses reported in this study were outlined in the clinical protocol, as approved by The Ohio State University Institutional Review Board (IRB) and in accordance with the principles expressed in the Declaration of Helsinki. Patient demographics and disease characteristics, as well as clinical outcomes and PK results, were reported previously [17]. DNA was obtainable from peripheral blood mononuclear cells (PBMCs) from 35 of the 52 patients on study. Demographics, baseline labs and disease characteristics for these patients are presented in Table 1.

**Table 1:** Demographics and baseline labs of patients included in pharmacogenetic analyses (n = 35).

Demographics			
GenderMaleFemale		269	
		Mean (Median)	Range
Age	years	61 (60)	39–84
Weight	kg	82 (82)	51.8–120.7
Body Surface Area	m^2^	1.9 (1.9)	1.54–2.41

Baseline CBC and Labs			
Platelets	×10^9^/L	112 (110)	16–284
White Blood Cells	×10^9^/L	75 (23)	3–314.5
Hemoglobin	mM	10 (10)	5.8–14.8
Albumin	g/dL	3.4 (3.3)	2.8–4.7
Protein	g/dL	5.6 (5.5)	4.5–7.3
Calcium	mg/dL	8.1 (8.2)	5.8–8.9
Chloride	mM	102 (102)	91–111
Potassium	mM	4.0 (3.9)	3.2–5.0
Magnesium	mg/dL	2.1 (2.0)	1.6–2.6
Sodium	mM	139 (139)	136–144
Phosphate	mg/dL	5.6 (4.5)	2.3–42.8
Alkaline Phosphatase (ALP)	U/L	91 (81)	50–245
Alanine Aminotransferase (ALT)	U/L	22 (20)	4.5–74
Aspartate Aminotransferase (AST)	U/L	57 (33)	16–470
Bilirubin	mg/dL	0.73 (0.68)	0.39–1.52
Creatinine	mg/dL	1.1 (1.1)	0.7–1.8
Lactate Dehydrogenase (LDH)	U/L	534 (289)	146–5603
Uric Acid	mg/dL	3.6 (3.2)	0.7–7.3
Blood Urea Nitrogen	mg/dL	15 (14)	8–32
Glucose	mg/dL	146 (131)	91–271
Carbon Dioxide	meq/L	29 (29)	24–36

### Pharmacogenetics

Genes known from *in vitro* studies to specifically affect flavopiridol disposition include UGT1A1, UGT1A9, ABCC2 and ABCG2. These and an additional set of 52 other genes that code for metabolic enzymes and transporters commonly involved in drug disposition were evaluated for the presence of known polymorphisms by direct sequencing and with a high-throughput SNPlex assay (Applied Biosystems) [32]. Genomic DNA was extracted from patient PBMCs and used to sequence promoter TATA box regions of UGT1A1 and UGT1A9 and specifically to identify the presence of the UGT1A1*28 and UGT1A9*22 polymorphisms. Primers for UGT promoter sequencing were as follows: 1A1-forward, 5′ – GGAAGTACTTTGCTGTGTTCACTCAAG; 1A1-reverse, 5′ – AAGGGTCCGTCAGCATGACATCAA; 1A9-forward, 5′ – CTTAACATTGCAGCACAGGGCATGTT; 1A9-reverse, 5′ – CGTACTTGTGCTTTCCATTGAGCATGGG. All sequence data was deposited in GenBank. To validate sequence results of the TATA box regions, single nucleotide polymorphism (SNP) specific primers were used in a fluorescence/gel-migration assay. Polymorphism-specific primers were utilized in the SNPlex assay to identify the presence of 189 other SNPs in drug metabolizing enzymes and transporters. A complete list of the genes and SNPs evaluated was presented previously [32].

### Population Pharmacokinetic Modeling and Validation

A population PK (popPK) model for 50 patients on study was previously reported [17]. This model served as the starting point to evaluate SNPs and other covariates for the subset of 35 patients with PGx data using NONMEM VI (Icon, Hanover, MD). With first order conditional estimation, a base structural model was built with proportional and additive residual error considering both between-subject variability (BSV) and between-occasion variability (BOV) on PK parameters, since PK data was available on two occasions from patients who received escalated doses.

Initial screening of the genetic data was completed with the allelic association test in HelixTree software (Golden Helix, Bozeman, MT) which produced unadjusted p-values for correlation with each of the base model-predicted PK parameters. Of the 189 SNPs (191 total including the UGT1A1*28 and UGT1A9*29 promoter polymorphisms from direct sequencing), 16 SNPs in 4 genes known previously to interact with flavopiridol (UGT1A1/9, ABCC2 and ABCG2), were retained for further analysis. From the remaining 175 total SNPs in 52 genes that were known to be involved in drug disposition, we filtered out from consideration SNPs with p-values>0.05 and minor allele frequencies less than 0.15. For direct fitting of selected polymorphisms to PK parameters, SNPs were converted to dichotomous categorical variables whereby the heterozygous category (M/m) for the major (M) and minor (m) alleles was combined with either the major or minor allele category as previously reported [33], [34].

Demographic, baseline laboratory covariates, and the total of 43 SNPs from above screening were then introduced with general additive modeling (GAM) and visual inspection of diagnostic plots using R v.2.9.0 and Xpose 4.0.4. These covariates identified by visual inspection and GAM as potentially meaningful (according to the Akaike information criterion, AIC) were introduced into the population model for further evaluation of the SNP associations. Using GAM, four sets of covariates including SNPs were retained for further evaluation in a full structural model.

For completeness, each individual covariate was fit to the structural model using the power (equation 1) or fractional change (equation 2) models,
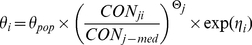
(1)

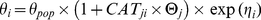
(2)where *θ_i_* is the individual PK parameter estimate, *θ_pop_* is the population parameter estimate for the typical individual, and *η_i_* is the sum of BSV and BOV parameters approximately log-normally distributed with standard deviations *ω* about a mean of 0. *CON_ji_* is the value of the continuous covariate *j* for individual *I*, *CON_j-med_* is the population median value of covariate *j* for all individuals, *CAT_ji_* is the value of the categorical covariate *j* for individual *i*, and *Θ_j_* is an estimated parameter. All covariates were evaluated in this way with the full 50-patient dataset, except for the SNPs which were evaluated after removal of individuals for whom no genetic data was available.

To identify a final multivariate model for all of the PK parameters simultaneously, we used selection methods that depend on changes in the objective function value. Using a cutoff of p≤.05, which corresponded to a minimum decrease of the objective function value (OFV) of 3.84 upon inclusion of each individual covariate based on the likelihood-ratio test, multivariate analysis with forward stepwise inclusion, backward stepwise deletion, and forward selection followed by backward elimination were applied to finalize the covariate model. Model selection in multivariate analysis was based on 1) minimum reduction of OFV by 3.84 (P≤0.05) for forward inclusion, 2) reduction of OFV by 6.64 or greater (P≤0.01) for backward deletion, and 3) decrease in residual error and/or BSV of the evaluated PK parameter. Interaction between covariates was examined by scatter plot of covariate values and change of OFV between models with single or combined covariates.

For bias evaluation the final model was fitted to replicate datasets using the bootstrap resampling technique in Wings for NONMEM [35], and PK parameter estimates and random effects for each of the replicate datasets were obtained. Two hundred replicate bootstrap datasets were generated and used for evaluation of parameter estimate precision. Model precision was evaluated by comparing mean parameter values and 95% bootstrap confidence intervals (CI) of the replicates with NONMEM outputs.

#### Cloning and Expression of SLCO1B1

The human SLC01B1 gene was isolated from the HEP-G2 cell line using methods similar to those previously published [36]. Briefly, RNA was extracted using Trizol Reagent and each half of the gene was PCR-amplified and cloned into the pcr-blunt II Topo vector (Invitrogen, Carlsbad, CA). The second half of the gene was digested with NotI and SnaBI and combined with the first half in the pcr-blunt II topo vector. The full length clone was then digested with KpnI and NotI and transferred into pcDNA 3.1 (+) (Invitrogen). Base pairs that were different from the reference sequence (NCBI Genbank ID, BC114376) were mutated using QuickChange (Stratagene, La Jolla, CA) via the manufacturer's protocols to match the reference and non-synonymous polymorphic variant sequences. Gene orientation and homology of reference, rs11045819 (T155P), rs2306283 (D130N), and rs4149056 (V174A) SLCO1B1 SNPs were confirmed through direct full length sequencing of clones prior to experimentation. A list of primers used for cloning, sequencing and mutagenesis (for introduction of nonsynonymous SNPs) is presented in Table 2.

**Table 2:** List of primers used for cloning, sequencing, and mutagenesis of SLCO1B1.

Cloning	
N-term, Forward (KpnI)	5′ GGG GTA CCA TGG ACC AAA ATC AAC ATT TGA AT 3′
N-term, Reverse	5′ GTT AGC CTT AGA TGA AGG CTG ACC 3′
C-term, Forward	5′ ACA AGT AAG CAG CTA TAT TGG TGC 3′
C-term, Reverse (NotI)	5′ GGG CGG CCG CTT AAC AAT GTG TTT CAC TAT CTG 3′

Sequencing	
N-term, Forward	5′ CAT GGA CCA AAA TCA ACA TTT G 3′
Middle, Forward	5′ GAT TGG TCC AAT CAT TG 3′
C-term, Forward	5′ GGG AAC CAG TCT GTG G 3′

Mutagenesis	
rs11045819, Forward	5′ CAC TCA ATA GAG CAT CAC CTG AGA TAG TGG G 3′
rs11045819, Reverse	5′ CCC ACT ATC TCA GGT GAT GCT CTA TTG AGT G 3′
rs2306283, Forward	5′ GAA ACT AAT ATC AAT TCA TCA GAA AAT TC 3′
rs2306283, Reverse	5′ GAA TTT TCT GAT GAA TTG ATA TTA GTT TC 3′
rs4149056, Forward	5′ CAT ACA TGT GGA TAT ATG CGT TCA TGG GTA ATA TGC TTC GT 3′
rs4149056, Reverse	5′ ACG AAG CAT ATT ACC CAT GAA CGC ATA TAT CCA CAT GTA TG 3′

#### Flavopiridol and Flavo-G Uptake Assays

Flavopiridol was obtained from the National Cancer Institute Cancer Therapy Evaluation Program. Flavopiridol-glucuronide (flavo-G) was extracted from patient urine and purified. Total urine through 24-hours after the start of flavopiridol dosing was collected from patients enrolled in an IRB-approved phase II protocol (NCI-7000). Octanol extraction followed by C-18 solid phase extraction was employed to isolate flavo-G from flavopiridol and other urine components. To quantify recovered flavo-G and verify purity, samples were incubated with β-Glucuronidase as previously described [19] and quantified via LC-MS/MS analysis with methods modified from those previously reported [37]. Purity was estimated at >95% via mass and UV chromatography.

Madin-Darby canine kidney (MDCK-II) and human embryonic kidney (HEK-293) cells, purchased from ATCC (Manassas, VA), were cultured in 5% CO^2^ at 37°C in Dulbecco's modified Eagle's medium supplemented with L-glutamine, 10% FBS, 100 units/ml penicillin, and 100 µg/ml streptomycin. Plates (24-well) were seeded with 2×10^5^ cells/well and transfected with the reference and polymorphic OATP1B1-containing vectors using FuGENE®6 Transfection Reagent per the manufacturer's protocols (Roche). Transfection efficiency and gene expression were evaluated with GFP vectors and real-time PCR, respectively. Forty-eight hours post-transfection, cells were dosed with 10 µM flavopiridol or flavo-G in OptiMEM® I (Invitrogen, Carlsbad, California) incubation media containing 4% bovine serum albumin for 10 and 30 minutes, respectively, at 37°C. After incubation, cells were washed with 4°C versene, trypsinized, and resuspended in 37°C versene at a total volume of 350 ul. A 150 µL aliquot of the cell suspension was lysed with 30 µl 6% Triton X-100 in PBS, and protein concentration was determined using Pierce® BCA protein assay (Thermo Scientific, Rockford, IL). The remaining 200µL cell suspensions were precipitated with 1mL, 4°C acetonitrile containing 200nM genistein, followed by vortex mixing and centrifugation at 16,000g for 10 min. The supernatant (1mL) was removed and dried in a vacuum concentrator then samples were resuspended in 150µL 95∶5 water∶acetonitrile plus 0.1% acetic acid, vortexed, and centrifuged. Supernatants (100 µL) were analyzed using liquid chromatography and mass spec conditions as described previously [38]. SN-38 (7-ethyl-10-hydroxycamptothecin, Sigma, St Louis, MO) and lenalidomide (obtained by extraction from donated patient capsules as previously reported [39]) were used as positive and negative control, respectively. Analytical methods for LC-MS/MS quantification lenalidomide was used as previously published [38]. For SN-38 LC-MS/MS quantification, a previously published method was modified and partially validated [40]. Calculated uptake velocities were normalized to total protein in each well, and results were compared against empty vector controls using Student's t-test.

#### Evaluation of Associations Between PGx, PK and Outcomes

To identify associations with genetics, the means of flavopiridol and flavo-G [17] PK parameters were compared based on SNP genotypes using Student's t-test and analysis of variance (ANOVA). For comparisons of PGx and clinical outcomes, SNP genotypes and response or toxicity grading was evaluated using Fisher's exact test. P-values were not further adjusted for multiple testing.

## Results

### Pharmacogenetic Analysis

DNA of adequate quality and quantity for analysis was available from 35 of the 52 patients treated on study, and both SNPlex and direct sequencing data were generated for these individuals (see Table 1 for demographics and pre-treatment characteristics of this patient subset). In addition to the 4 genes of interest, 17 genes and 27 SNPs met our criteria for further study (see Table 3). Among these, SLCO1B1 was selected for further evaluation given its known physiological relevance for a broad set of drugs and its potential role of transporting flavopiridol into liver for subsequent metabolism and excretion.

**Table 3:** List of 4 Candidate genes with 16 SNPs and 17 exploratory genes with 27 SNPs having minor allele frequencies (f)>0.15 and allelic association test p<0.05.

Original 16 Candidates		Exploratory (f>0.15, p<0.05)	
Name	ID	Name	ID
ABCC2	rs17222568	ABCA2	rs2271862
ABCC2	rs2273697	ABCB4	rs1202283
ABCC2	rs3740066	CYP1A2	GID3534
ABCC2	rs717620	CYP1B1	rs1056836
ABCC2	rs8187694	CYP2D6	GID2484
ABCG2	rs1564481	CYP2D6	GID2936
ABCG2	rs2231142	CYP2D6	GID2989
ABCG2	rs2622605	CYP2E1	GID1135
ABCG2	rs2622624	CYP3A4	m392A
ABCG2	rs3114018	CYP3A4	m747C
UGT1A1	*28 (TA)n	CYP3A4	GID13989
UGT1A1	1456T>G	CYP3A5	31611C
UGT1A1	247T>C	CYP3A5	GID6986
UGT1A1	686C>A	GSTM3	rs1537234
UGT1A1	rs4148323	GSTM3	rs1799735
UGT1A9	*22 (T)n	GSTM3	rs7483
		GSTP1	rs4891
		GSTP1	rs947894
		GSTT1	rs4630
		MDR1	GID3435
		MTHFR	rs1801133
		NAT2	GID481
		SLCO1B1	rs11045819
		SLCO1B1	rs2291075
		SLCO1B1	rs4149056
		SOD2	rs1799725
		SULT1A1	rs1042157

### PK Modeling

#### Base structural model

A total of 577 plasma concentration-time values from 50 of 52 patients in the clinical study were included for PK data analysis using two-compartmental kinetics with first-order elimination, as described previously [17]. Based on this previous analysis, BSV was initially assumed on each parameter in the model. However, removal of BSV for V1 did not significantly change OFV (increase of 1 unit). After removal of BSV on V1, addition of BOV on single or multiple parameters was tested in the model. Addition of BOV on clearance (CL) resulted in the most significant change in OFV. The final base model thus included BSV on CL, inter-compartmental clearance (Q) and volume of peripheral compartment (V2), and BOV on CL. Base model parameter estimates and random effects are presented in Table 4.

**Table 4:** Base model PK parameter estimates.

Model Term	Parameter	Estimate (CV%)	BSV (η) (CV%)	BOV (CV%)
Full Dataset				
CL = θ_CL_·exp(BSV_CL_+BOV_CL_)	θ_CL_ (L/h)	31.6 (4.6)	20.9 (44.9)	22.1 (35.2)
V1 = θ_V1_	θ_V1_ (L)	65.4 (2.8)		
Q = θ_Q_·exp(BSV_Q_)	θ_Q_ (L/h)	8.22 (10.0)	63.6 (27.9)	
V2 = θ_V2_·exp(BSV_V2_)	θ_V2_ (L)	145 (14.6)	76.6 (34.4)	
Reduced Dataset				
CL = θ_CL_·exp(BSV_CL_+BOV_CL_)	θ_CL_ (L/h)	30.9 (5.0)	17.0 (71.9)	22.6 (40.4)
V1 = θ_V1_	θ_V1_ (L)	64.9 (3.4)		
Q = θ_Q_·exp(BSV_Q_)	θ_Q_ (L/h)	8.21 (11.6)	57.6 (33.1)	
V2 = θ_V2_·exp(BSV_V2_)	θ_V2_ (L)	142 (14.6)	67.0 (42.8)	

The full (56 subjects, 577 plasma concentrations) and reduced (35 subjects, 388 plasma concentrations) datasets were used. Parameters: CL, clearance; V1, volume of central compartment; Q, inter-compartmental clearance; V2, volume of peripheral compartment (units are noted in parenthesis). BSV and BOV are listed as %CV. Θ, typical value of the PK parameters; BSV, between-subject variability; BOV, between-occasion variability.

#### Covariate model

**Figure 1:**
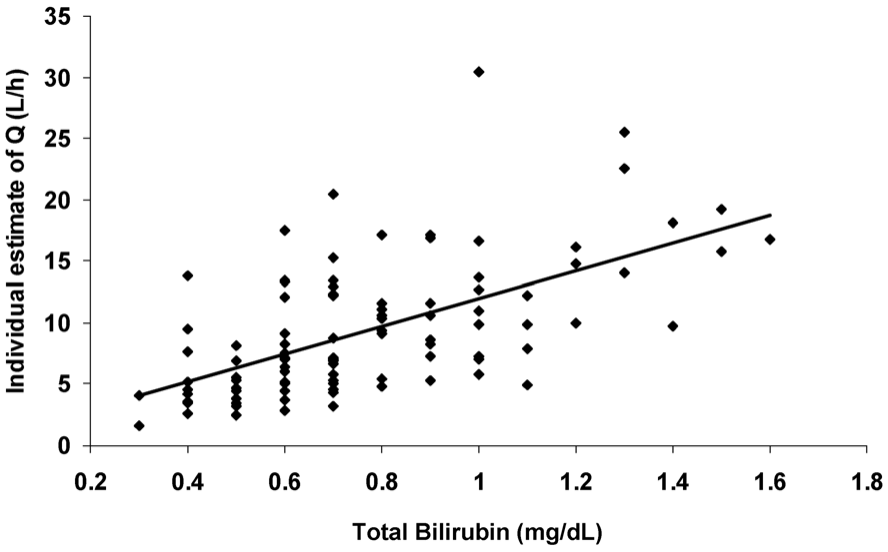
Individual estimate of Q vs. baseline total bilirubin (with full phase I dataset). Regression analysis; Q = 11.21 * Bilirubin level+0.7422 (P<0.01).

Demographic and lab covariates from GAM screening were subsequently evaluated in the base model with univariate analysis (see Table 5). With the modified random error in the base model, bilirubin was indicated as the most significant covariate with a direct positive effect on Q. Addition of bilirubin decreased OFV by 8.07 and BSV (on Q) from 63.64% to 55.41% (see Figure 1).

**Table 5:** Six significant covariate-parameter correlations after inclusion of single demographic or lab covariates.

Demographic or Lab Covariate	Parameter	Δ OFV	THETA of Covariate (CV%)
Bilirubin level (BIL)	Q	−8.07	0.843 (28.6)
Age (AGE)	V2	−6.348	−1.96 (41.3)
Platelet level (PLT)	CL	−4.925	−0.179 (54.3)
White blood cells count (WBC)	V1	−4.428	−0.061 (44,2)
Blood urea nitrogen (BUN)	CL	−4.34	0.164 (57.2)
Alanine Aminotransferase (ALT)	V2	−3.878	0.448 (34.4)

Comparison of OFV is made between model with single covariate and the base model. Δ OFV, change in objective function value.

**Figure 2:**
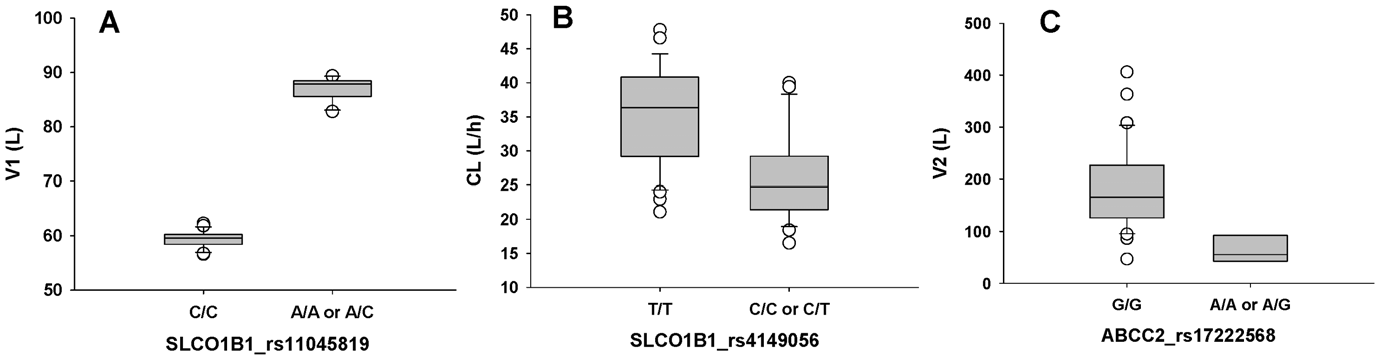
Relationships between the most significant genetic covariates and estimates for each of the 4 PK parameters from univariate analysis in the phase I dataset. Box plots represent the scatter of individual V1 estimates vs. SLCO1B1 rs11045819 (Panel A), CL estimates vs. SLCO1B1 rs4149056 (Panel B), and V2 estimates vs. ABCC2 rs8187710 (Panel C). For rs11045819, data is displayed with V1 estimates using BSV on V1.

To evaluate genetic covariates, the dataset was reduced by removing patients for whom no genetic data was available. This reduced the dataset from 50 to 35 subjects and from 577 to 388 records. Univariate analysis on genetic covariates with this dataset identified the 14 most significant covariate-parameter relationships (see Table 6). Using the selection methods discussed above, we retained in all of the final models SLCO1B1 rs11045819 and ABCC2 rs8187710. The relationships between these SNPs and their respective base model-estimated PK parameters are displayed in Figure 2.

**Table 6:** Significant covariate-parameter correlations after inclusion of single genetic covariates.

Genetic Covariate	Parameter	Δ OFV	THETA of Covariate (CV%)
SLCO1B1_rs11045819	V1	−18.845	0.426 (35.9)
SLCO1B1_rs4149056	CL	−13.144	−0.259 (28.3)
ABCC2_rs2273697	V2	−12.164	2.27 (66.1)
UGT1A9*22	V1	−9.462	0.244 (41.8)
ABCC2_rs8187710	V2	−7.542	−0.647 (23.3)
UGT1A1*28	CL	−5.917	−0.224 (55.4)
ABCC2_rs8187710	V1	−5.681	−0.204 (73.5)
SLCO1B1_rs2291075	V1	−5.528	0.191 (51.3)
SLCO1B1_rs2291075	CL	−5.28	−0.211 (34.4)
ABCC2_rs717620	Q	−4.781	0.692 (61.6)
ABCG2_rs3114018	V1	−4.721	−0.191 (53.4)
SLCO1B1_rs3829310	V1	−4.345	−0.168 (66.7)
ABCC2_rs717620	CL	−4.166	0.201 (58.7)

Comparison of OFV was made between models with single genetic covariates and the base model, using the reduced dataset with deletion of subjects having missing values on each individual genetic covariate. Δ OFV, change in objective function value.

### Functional Validation of SCL01B1's Role in Flavopiridol and Flavo-G Uptake

**Figure 3:**
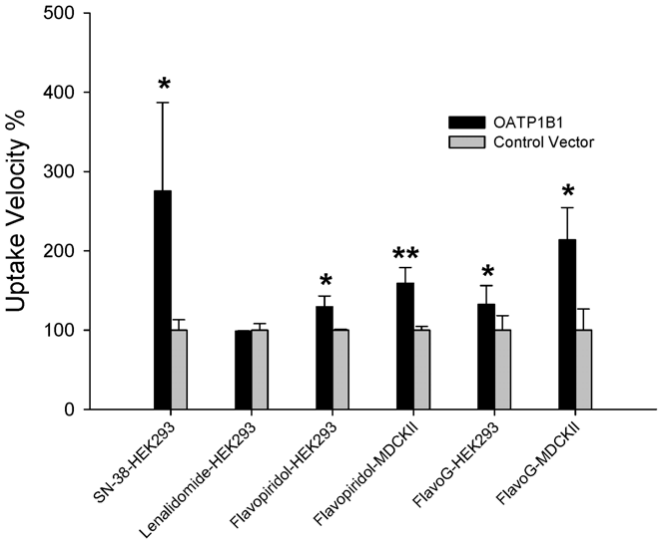
Uptake of flavopiridol, flavo-G, SN-38 and lenalidomide in OATP1B1 transfected cells. The bar graph indicates means + SD for triplicate determinations of 10 µM flavopiridol, flavo-G, SN-38 and lenalidomide uptake rates in cell lines (HEK-293 or MDCK-II) transfected with empty control vector or vector cloned with the OATP1B1 gene (SCLO1B1). Incubations with flavo-G were for 30 min., and all other drugs were for 10 min. Transport rates are expressed as percentages normalized to empty vector control. * p<.05, ** p<0.001,Student's t-test.

Although associations between PK and SNPs in ABCC2 may have been expected due to known *in vitro* interactions of MRP2 and flavopiridol, no such evidence existed prior to this study for the role of SLCO1B1/OATP1B1 in flavopiridol transport. To determine if the observed associations between flavopiridol PK and SLCO1B1 PGx were functionally relevant for flavopiridol disposition, we measured uptake of flavopiridol and flavo-G in cells transfected with SLCO1B1. Transfection efficiencies were estimated at approximately 60% using GFP-containing control vectors. Mean uptake velocities were 261±12 fmol/mg protein/10 min and 38±10 fmol/mg protein/30 min for flavopiridol and flavo-G, respectively, in MDCK-II cells. Flavopiridol transport rates in HEK-293 cells were approximately 2–3 fold higher than in MDCK-II cells suggesting its transport may be affected by the different membrane and transporter compositions in the two cell lines. Flavo-G transport rates were similar in both cell lines. Figure 3 shows normalized uptake velocities of flavopiridol and flavo-G in both HEK293 and MDCK-II cells transfected with either SLCO1B1 or empty vector. Expression of the transfected SLCO1B1 gene was verified with real-time PCR (data not shown). Functional expression of OATP1B1 was verified by evaluating uptake of a positive control substrate, SN-38 [41]. A second agent, lenalidomide, was used as a negative control substrate. Total intracellular accumulation and Initial transport velocities of SN-38, flavopiridol, and flavo-G were significantly increased in HEK293 and MDCK-II cells transiently transfected with SLCO1B1, compared to empty control vectors, whereas no increased uptake was shown for lenalidomide.

**Figure 4:**
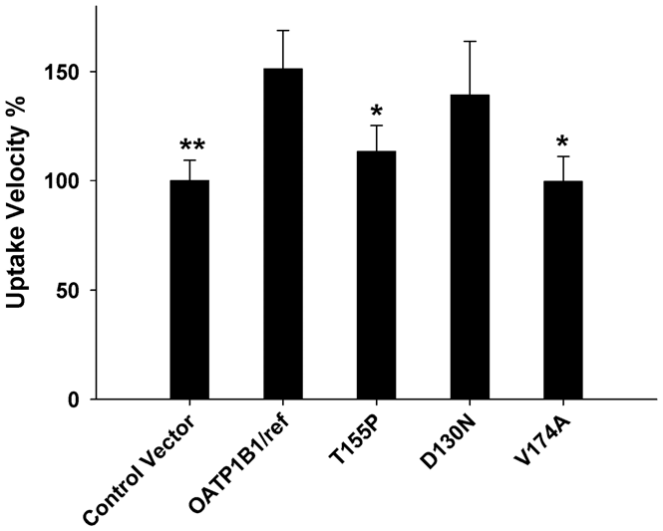
Uptake of flavopiridol in nonsynonymous polymorphic variants of OATP1B1. The bar graph indicates means + SD for triplicate determinations of 10 µM flavopiridol uptake rates in MDCK-II cells transfected with empty control vector or vector cloned with the reference or polymorphic SLCO1B1 genes. All incubations were for 10 min. Variants are indicated with respect to their amino acid change; T155P = rs11045819, D130N = rs2306283, V174A = rs4149056. Transport rates were normalized to empty vector control. Differences in uptake rates were compared to the reference OATP1B1 transporter. * p<.05, ** p<0.01, Student's t-test.

We further evaluated the uptake of flavopiridol in MDCK-II cells transfected with the SLCO1B1 polymorphic variants with amino acid changes relative to the reference sequence (i.e. nonsynonymous SNPs). These included rs11045819 (T155P), rs2306283 (D130N), and rs4149056 (V174A). The results indicated significant decreases in flavopiridol transport rates (t-test p-value<0.05) for the rs11045819 and rs4149056 variants, but the transport rate of the rs2306283 polymorph was similar to that in the reference SLCO1B1 transporter. Figure 4 displays these results.

### Final PK Model Determination

To arrive at a final model with significant PGx, lab and demographic covariates, the reduced data set was used to reevaluate the demographic and lab covariates retained after univariate analysis with the full dataset (see Table 6). Bilirubin effect on Q was apparent and remained the most significant demographic or lab covariate with the reduced dataset. However, significant effects of blood urea nitrogen on CL and alanine aminotransferase on V2 that were observed in the full dataset disappeared with use of the reduced dataset. The remaining four significant demographic or lab covariates and the two significant SNPs (SLCO1B1 rs11045819 and ABCC2 rs8187710) were then evaluated by forward addition and backward deletion. Only the two SNPs and bilirubin were retained as significant covariates with the reduced dataset. Relative to the base model, the final model displayed an OFV reduction of 34.11. Table 7 lists the final model parameter estimates.

**Table 7:** Final model parameter estimates.

Parameter	Estimate (CV%)	BSV (CV%)	BOV (CV%)
θ_CL_ (L/h)	30.9 (5.6)	17.8 (89.9)	23.3 (44.4)
θ_V1_ (L)	60.6 (4.1)		
θ_Q_ (L/h)	8.5 (11.5)	57.4 (34.2)	
θ_V2_ (L)	181 (15.4)	60.5 (44.0)	
θ_SLCO1B1_rs11045819_ (on V1)	0.417 (34.1)		
θ_BIL_ (on Q)	0.939 (37.2)		
θ_ABCC2_rs8187710_ (on V2)	−0.68 (22.9)		
residual error	35.4 (8.2)		

The reduced dataset with 35 subjects and 388 plasma concentrations was used. Parameters: CL, clearance; V1, volume of central compartment; Q, intercompartmental clearance; V2, volume of peripheral compartment (units are noted in parenthesis). BSV and BOV are listed as %CV. Θ, typical value of the PK parameters; BSV, between-subject variability; BOV, between-occasion variability.

The bootstrap method was used to assess bias in the final covariate model. From the reduced data set, 200 replicate data sets were generated and used for the evaluation of the stability of the final covariate model. Table 8 lists the results of the bootstrap procedure, presented as mean and 95% bootstrap confidence intervals of the parameter estimates and random effects of the final model. Mean estimated parameter values from the bootstrap were within 11% of the parameter estimates of the original data set indicating reliability in the developed model [42].

**Table 8:** Population estimates from final model and bootstrap analysis.

Parameter	Final model estimate (%CV)	Bootstrap summary			
		Mean	Relative bias (%)	Lower 95% CI	Upper 95% CI
θ1 (CL) (L/hr)	30.9 (5.1)	31.30	0.65	27.98	34.62
θ2 (V1) (L)	60.6 (4.3)	60.70	−0.17	54.60	66.79
θ3 (Q) (L/hr)	8.5 (11.5)	8.29	−2.59	6.30	10.28
θ4 (V2) (L)	181 (15.6)	170.31	−4.42	123.31	217.31
θ5 (SLCO1B1_rs11045819) (on V1)	0.42 (30.7)	0.44	0.95	0.21	0.67
θ6 (BIL) (on Q)	0.94 (36)	0.84	−10.96	−0.01	1.70
θ7 (ABCC2_rs8187710) (on V2)	−0.68 (16.5)	−0.61	−6.18	−0.98	−0.24
OMEGA1 (CL)	0.18 (64)	0.16	−5.23	0.05	0.28
OMEGA2/3 (IOV-CL)	0.23 (34.7)	0.23	1.41	0.15	0.31
OMEGA4 (Q)	0.57 (36.4)	0.52	−5.52	0.24	0.80
OMEGA5 (V2)	0.6 (38)	0.56	−5.57	0.29	0.84

Relative bias,i(%) = (Pbs,i−Pest,i)/Pest,I×100; Pbs,i: mean of parameter i estimate from bootstrap; Pest,i: final parameter i estimate.

### Evaluation of Flavo-G and Bilirubin vs. PGx

**Figure 5:**
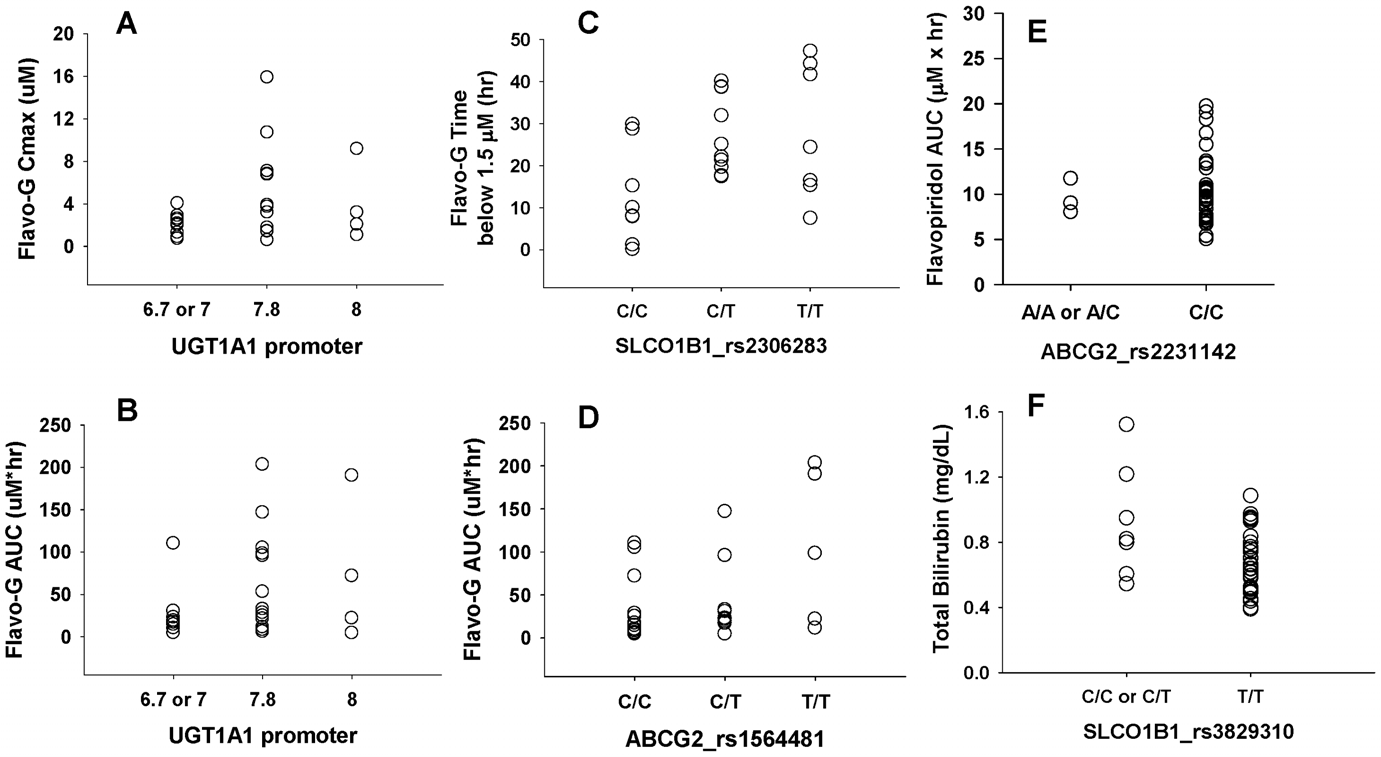
Association of PGx, PK and bilirubin from the phase I dataset. Flavo-G PK parameter estimates from 27 patients were evaluated for relationships with PGx. Fewer TA repeats in the UGT1A1 promoter were weakly associated with lower flavo-G Cmax (Panel A) and AUC (Panel B). For SLCO1B1, the rs2306283 SNP correlated with flavo-G plasma concentrations (the total time flavo-G plasma concentrations were below 1.5 µM, p = .019; Panel C, one outlier removed). For ABCG2, the rs1564481 SNP was associated with flavo-G AUC (p = .050; Panel D) and the rs2231142 SNP was associated with flavopiridol AUC (p = 0.08, Panel E). The SLCO1B1 rs3829310 minor C allele was associated with higher baseline total bilirubin (p = .011; Panel F). For plots A and B, the x-axis indicates the number of promoter TA repeats for both gene copies (6.7 = 6 and 7 TA repeats; 7 = 7 TA repeats for each gene copy; 7.8 = 7 and 8 TA repeats; 8 = 8 TA repeats for each gene copy).

Flavo-G PK parameter estimates reported previously in 27 pts on study [17] were evaluated to identify PGx associations. The trends observed indicated that fewer TA repeats in the UGT1A1 promoter were weakly associated with lower flavo-G Cmax (2.17+/−0.99 vs. 5.08+/−4.12 µM) and AUC (26.43+/−30.26 vs. 66.16+/−64.37 hr* µM) (p = .057 and .077, respectively). Only two transporter SNPs were associated with flavo-G PK. The SLCO1B1 rs2306283 SNP correlated with flavo-G plasma concentrations (the total time in hours flavo-G concentrations were below 1.5 µM, p = .019), and the ABCG2 rs1564481 SNP was associated with flavo-G AUC (p = .050). We also evaluated flavopiridol AUC with respect to PGx, and one trend was observed with the ABCG2 rs2231142 SNP (p = 0.08). Bilirubin levels were not associated with SNPs in UGT1A1, but the SLCO1B1 rs3829310 minor C allele was associated with higher baseline bilirubin (p = .011). These relationships are presented in Figure 5.

### Correlations of PGx and Outcomes

To determine if the presence of polymorphisms was significantly associated with outcomes, clinical response and the primary toxicities (TLS, diarrhea and CRS) were evaluated against PGx. Results indicated SNPs in SLCO1B1 and ABCG2, but not in UGT1A1/9 or ABCC2, were associated with response. SNPs significantly associated with improved response included the SCLO1B1 rs11045819 (CC genotype, p = .041) and ABCG2 rs1564481 (T allele, p = .037). The SLCO1B1 rs2306283 SNP alone did not meet the significance criteria (T allele, p = .061), but the combination of this and the SLCO1B1 rs11045819 SNP was significant (p = .007). No SNPs met the significance criteria when compared against TLS, although the most closely associated SNP was SLCO1B1 rs4149056 (C allele, p = .056). Similaraly, the most closely associated SNPs with diarrhea and CRS were SCLO1B1 rs2306283 (T allele, p = .055) and ABCG2 rs1564481 (T allele, p = .074).

### Validation of findings with a Phase II Study

To evaluate the validity of the findings from the 35-patient dataset, a second dataset was evaluated for associations between PGx and PK. The validation set comprised data from 66 CLL patients who were treated with the same flavopiridol dosing regimen in a separate phase II study (NCI-7000, NCT00098371). As with the phase I study, enrolled patients provided informed written consent, and plasma and PBMC samples were obtained according to The Ohio State University IRB approved protocol. Clinical results of this study were reported previously [43]. Plasma and DNA samples from this study were analyzed using the methods described above to generate flavopiridol and flavo-G concentration-time data, PK parameter estimates, and PGx data for each patient. Covariates found to be significantly associated in univariate analysis with the phase I dataset, including demographic and baseline laboratory covariates and SNPs in UGT1A1, ABCC2, ABCG2 and SLCO1B1, were compared with the phase II pharmacokinetic data.

**Figure 6:**
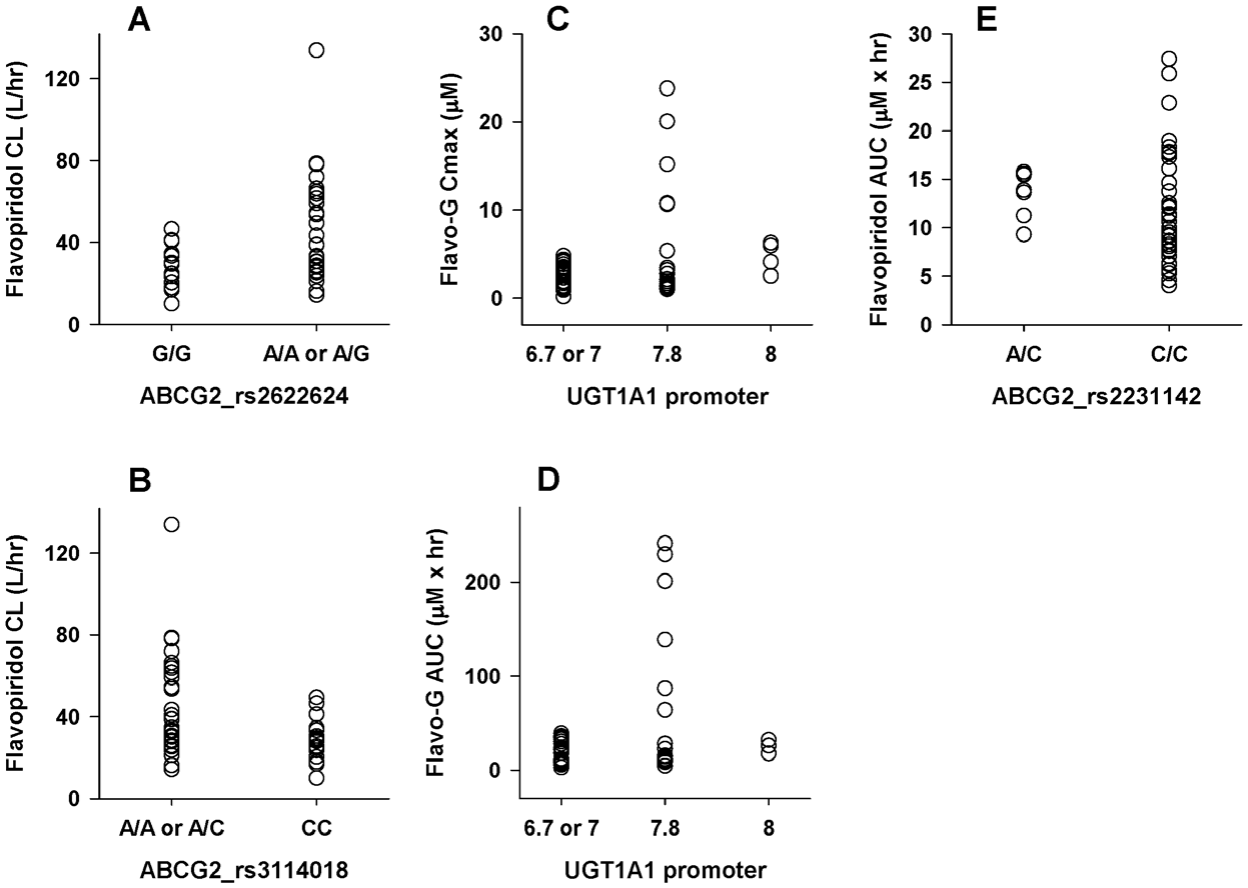
Association of PGx and PK from the phase II dataset. Flavopiridol and flavo-G PK parameter estimates from the phase II study was evaluated for relationships with PGx. Presented are flavopiridol CL associations with ABCG2 rs2622624 (p = 0.008, Panel A) and rs3114018 (p = 0.004, Panel B); UGT1A1*28 (promoter) polymorphisms associated with flavo-G Cmax (p = 0.02, Panel C) and AUC (p = 0.04, Panel D); the ABCG2 rs2231142 SNP was weakly associated with flavopiridol AUC (Panel E, p = .08); For plots C and D, the x-axis indicates the number of promoter TA repeats for both gene copies (6.7 = 6 and 7 TA repeats; 7 = 7 TA repeats for each gene copy; 7.8 = 7 and 8 TA repeats; 8 = 8 TA repeats for each gene copy).

Significant associations and trends were observed with the validation dataset. For flavopiridol PK, these included significant associations between the SCLO1B1 rs2306283 SNP and Q (p = 0.02) and between both the ABCG2 rs2622624 and rs3114018 SNPs and CL and V1 (rs2622624, p = 0.008 and 0.04; rs3114018, p = 0.004 and 0.006 for CL and V1, respectively). The SLCO1B1 rs3829310 SNP was weakly associated with flavopiridol CL and AUC (p = 0.08 and 0.08, respectively). The ABCG2 rs2231142 SNP showed a similar trend with AUC (p = .08). For flavo-G, significant associations included Cmax and AUC with the UGT1A1*28 promotor SNP (p = 0.02 and 0.04, respectively). Bilirubin levels were significantly associated with the SLCO1B1 rs3829310 and SLCO1B1 rs2291075 SNPs (p = 0.02 and p = 0.04, respectively). Figure 6 displays a subset of these relationships.

## Discussion

Safe and effective use of flavopiridol will require a thorough understanding of the factors influencing the onset of hyper-acute tumor lysis syndrome and inter-individual variability in response. We previously reported that at least some of the variability in outcomes may be explained by PK, yet significant portions of this variability remain unexplained. In this first evaluation of pharmacogenetics using the flavopiridol PK-derived schedule, we identified a novel flavopiridol-transporter interaction and confirmed its functional relevance *in vitro*. Furthermore, a subset of the polymorphisms evaluated in this study was significantly associated with outcomes. The striking activity of this dosing schedule in CLL and flavopiridol's emerging activity with combination chemotherapy in other diseases demand these pharmacogenetic and other factors be identified and further characterized.

To expand on our previous evaluation of flavopiridol using the PK-directed schedule, we sought to characterize the role of PGx in flavopiridol disposition. We combined focused and exploratory approaches, whereby PK data from a phase I dataset was evaluated against SNPs in candidate genes previously shown to metabolize or transport flavopiridol in *in vitro* studies, as well as SNPs in genes not known to interact with flavopiridol. The candidate genes evaluated included ABCC2, ABCG2, UGT1A1 and UGT1A9. Indeed, at least one SNP in each of these genes was significantly associated in univariate analysis with flavopiridol and flavo-G PK. In multivariate analysis with the phase I dataset, a single SNP in ABCC2 (rs8187710) remained significant. This is a non-synonymous SNP resulting in a C1515Y alteration and is part of a haplotype with the rs17222723 SNP (also a non-synonymous SNP with a V1188E residue change). This haplotype has been reported to be associated with PBMC accumulation of lopinavir [44], susceptibility to nonalcoholic fatty liver disease [45], and doxorubicin-induced cardiotoxicity [46]. This is a relatively infrequent SNP, and only 5 of 35 and 4 of 51 individuals were genotyped for the polymorphic A allele in the phase I and II datasets, respectively. Significant associations between this SNP and PK parameters were not observed in the phase II dataset.

Although UGT1A1 SNPs were not included in the final popPK model, both flavopiridol and flavo-G were correlated with the UGT1A1*28 promoter polymorphism, and fewer TA repeats (6 and 7) were associated with lower flavo-G concentrations and AUC in both the phase I and II datasets. One of the most broadly studied substrates of UGT1A1 and 1A9 is irinotecan/SN-38, which shares metabolic pathways and has a similar toxicity profile (diarrhea) with flavopiridol. Interestingly, the *3 polymorphism of 1A9 was previously shown to be associated with SN-38 but not flavopiridol disposition [30]. Innocenti and colleagues measured ratios of flavo-G∶flavopiridol and identified an indirect relationship between flavo-G levels and diarrhea [31]. Zhai and colleagues reported PK and UGT1A1 polymorphism data in 49 patients with refractory neoplasms treated with 1-hour IV flavopiridol [29]. However, their results indicated no correlation of the TATA box promoter UGT1A1*28 genotype with flavopiridol PK or diarrhea severity. While we did not identify correlations with diarrhea for flavopiridol and flavo-G PK [17], we observed one association with PGx and diarrhea.

The 35-patient dataset evaluated in this study was small with regard to exploratory PGx, and therefore an additional dataset from 51 patients was used to evaluate the validity of the findings. While the associations identified in each dataset were not identical, similar trends were observed between PK parameters and SNPs in the candidate genes. Furthermore, the clinical associations we observed with SLCO1B1 were functionally validated *in vitro*. SLCO1B1 is important for the disposition of statin drugs [47] and various anti-cancer agents including irinotecan [48]. It was recently highlighted by the International Transporter Consortium as one of the seven most relevant transporters for drug development due to its broad substrate specificity, potential for drug-drug interactions, and clinically relevant polymorphisms [49]. Importantly, functional transport data indicated this gene may play a role in hepatic uptake of both flavopiridol and flavo-G. All five of the SLCO1B1 SNPs evaluated in the phase I dataset were significant in univariate analysis, and two of these (rs11045819 on V1 and rs4149056 on CL) were the strongest associations observed of any covariates evaluated for flavopiridol PK in multivariate analysis. These are non-synonymous SNPs affecting the amino acid sequence in OATP1B1. Previous studies indicated these variants were associated with reduced uptake activity of the OATP1B1 substrates estrone-3-sulfate, estradiol-17β-d-glucuronide, atorvastatin, cerivastatin, pravastatin, the SN-38 metabolite of irinotecan and rifampicin in [50]–[51]. These reports are consistent with our *in vitro* data which indicated these SNPs significantly reduced flavopiridol transport, while the other nonsynonymous SNP evaluated (rs2306283) had no measureable effect. Interestingly, this was the only SLCO1B1 SNP significantly associated with flavo-G PK. A recently reported study in MDCK-II cells with this SNP indicated increased transport of bromosulfophthalein and decreased transport of cholyltaurine [51]. Collectively, the data may suggest that the altered protein sequence affects OATP1B1 transport of flavopiridol and flavo-G differently. Further *in vitro* evaluations of these and other SNPs will be required to characterize their functional impact with respect to flavopiridol and flavo-G disposition. Additionally, the data presented suggests flavopridol may be a weak substrate of SLCO1B1 in comparison with SN-38 under the evaluated experimental conditions. However, characterization of the kinetics of flavopiridol and flavo-G transport via SLCO1B1 will be necessary for understanding the full impact this gene has on overall flavopiridol disposition.

The observed associations between PGx and outcomes in this study are encouraging as potential indicators for patient response. While no SNP met the significance criteria for association with TLS, the polymorphism most closely associated with this toxicity was SLCO1B1 rs4149056 (ANOVA p = .056). The strength of the associations between PK, outcomes and multiple SNPs in SLCO1B1 and the confirmation of functional OATP1B1 transport of both flavopiridol and flavo-G support the potential for this gene to be clinically relevant in patients receiving flavopiridol treatment. Overall, analysis of the phase II dataset revealed significant associations with the candidate genes that were supportive of the findings from the phase I dataset. In particular, trends in flavopiridol AUC with respect to the ABCG2 rs2231142 SNP and flavo-G Cmax and AUC with respect to the UGT1A1*28 polymorphism were strikingly similar between the two datasets (see Figures 5 and 6). Again, further validation of these associations will be essential in larger datasets.

This work modifies and develops further our previously reported population model for the PK-directed schedule of flavopiridol. We demonstrate the most significant covariates in the phase I dataset to be polymorphisms in two transporter genes, SCLO1B1 and ABCC2, and the phase II validation set revealed significant associations with polymorphisms in ABCG2. While larger data sets are necessary to fully characterize the clinical impact of polymorphisms in SLCO1B1 and other genes on flavopiridol PK, our findings are supportive of a clinically significant role for PGx in flavopiridol disposition. The composite data to date suggests inter-individual variability in outcomes from flavopiridol therapy will be due to a combination of pharmacokinetics, pharmacogenetics and potentially other factors related to tumor cell sensitivity to flavopiridol's cytotoxic effects. Inter-individual variability is of particular concern for chemotherapeutic drugs with relatively narrow therapeutic windows. The developing model for flavopiridol PK may ultimately help to explain this variability by incorporating pharmacogenetic and other significant factors. With sufficient data and validation, this model may ultimately serve as a tool for predicting PK and associated outcomes in individuals prior to therapy. If sufficiently robust, such a tool would be clinically useful in identifying individuals likely to respond and/or experience severe TLS or other toxicities upon receiving flavopiridol. As a phase II registration study of flavopiridol in relapsed CLL nears completion and additional combination studies in hematologic and solid tumor diseases begin accruing patients, further exploration and characterization of the factors influencing inter-individual variability in outcomes from therapy will be necessary to insure the broad and safe clinical use of this drug.
